# Androgen Receptor-Directed Molecular Conjugates for Targeting Prostate Cancer

**DOI:** 10.3389/fchem.2019.00369

**Published:** 2019-05-28

**Authors:** Giovanni L. Beretta, Nadia Zaffaroni

**Affiliations:** Molecular Pharmacology Unit, Department of Applied Research and Technological Development, Fondazione IRCCS Istituto Nazionale dei Tumori, Milan, Italy

**Keywords:** prostate cancer, drug resistance, drug conjugates, proteolysis targeting chimeras, selective androgen receptor degraders

## Abstract

Due to its central role in the cellular biology of prostate cancer (PC), androgen receptor (AR) still remains an important therapeutic target for fighting this tumor. Several drugs targeting AR have been reported so far, and many new molecules are expected for the future. In spite of their antitumor efficacy, these drugs are not selective for malignant cells and are subjected to AR-mediated activation of drug resistance mechanisms that are responsible for several drawbacks, including systemic toxicity and disease recurrence and metastasis. Among the several strategies considered to overcome these drawbacks, very appealing appears the design of hybrid small-molecule conjugates targeting AR to drive drug action on receptor-positive PC cells. These compounds are designed around on an AR binder, which selectively engages AR with high potency, coupled with a moiety endowed with different pharmacological properties. In this review we focus on two classes of compounds: a) small-molecules and AR-ligand based conjugates that reduce AR expression, which allow down-regulation of AR levels by activating its proteasome-mediated degradation, and b) AR-ligand-based conjugates for targeting small-molecules, in which the AR binder tethers small-molecules, including conventional antitumor drugs (e.g., cisplatin, doxorubicin, histone deacetylase inhibitors, as well as photo-sensitizers) and selectively directs drug action toward receptor-positive PC cells.

## Introduction

Prostate cancer (PC) is the leading cause of tumor death in men of Western countries and the malignancy is increasing in developing nations (Jemal et al., 2011). Androgen receptor (AR) represents a pivotal driving force in the development and progression of PC. Upon stimulation with androgens, AR translocates to the nucleus, where it binds to thousands of sites throughout the human genome to regulate transcription of responsive genes, many of which are involved in the control of crucial cellular functions such as growth and proliferation. In addition, AR has an impact on prostate cancer development also by affecting genomic stability and DNA repair (Mills, 2014). Early diagnosed patients are treated with surgery or radiotherapy, which fail in 10–20% of cases. Recurrent patients are exposed to androgen deprivation therapy (ADT). However, ADT efficacy is time-limited and most patients undergo disease progression and develop castration-resistant prostate cancer (CRPC) (Watson et al., 2015). These tumors are often still dependent on AR and continue to growth in presence of very low levels of circulating androgens (Huang et al., 2018). In spite of the antitumor efficacy demonstrated by antiandrogen drugs (e.g., bicalutamide, flutamide, ARN-509, enzalutamide), the emergence of AR resistance mechanisms, including (a) AR gain-of-function mutations with increased sensitivity to androgens or increased recruitment of AR co-activators; (b) AR amplification/over-expression; (c) androgen independent AR activation; (d) expression of constitutively active AR splice variants; (e) intratumoral conversion of adrenal androgens and androgen production, are responsible for treatment failure (Guerrini et al., 2014; Ferroni et al., 2017; Howard et al., 2018; Huang et al., 2018; Paschalis et al., 2018). In this scenario, the development of new drugs represents a critical need, and novel therapies for PC are emerging (Sonnenburg and Morgans, 2018). Among these, the rational design of multivalent conjugates carrying a moiety that interacts with AR (AR binder) coupled with a residue endowed with antitumor activity represents a intriguing strategy for targeting malignant PC without affecting normal cell viability. The rational engineering of hybrid small-molecules is aimed not only at bypassing drug resistance, but also to selectively target tumor cells and, consequently, to reduce systemic toxicity. Here we focus on two classes of compounds: a) small-molecules and AR-ligand based conjugates that reduce AR expression, which allow down-regulation of AR levels by activating its proteasome-mediated degradation and b) AR-ligand based conjugates for targeting small-molecules, in which the AR binder tethers small-molecules, including conventional antitumor drug (e.g., cisplatin, doxorubicin, histone deacetylase inhibitors, as well as photo-sensitizers) and selectively directs drug action toward receptor-positive PC cells.

## Small-Molecules and AR-Ligand Based Conjugates that Reduce AR Expression

Selective AR degraders (SARD) and PROteolysis Targeting Chimeras (PROTAC) are two classes of compounds endowed with antitumor activity on hormone-refractory PC that knock-down AR (wt and mutant) content via proteasome-mediated degradation.

### Selective AR Degraders (SARD)

**Figure 1:**
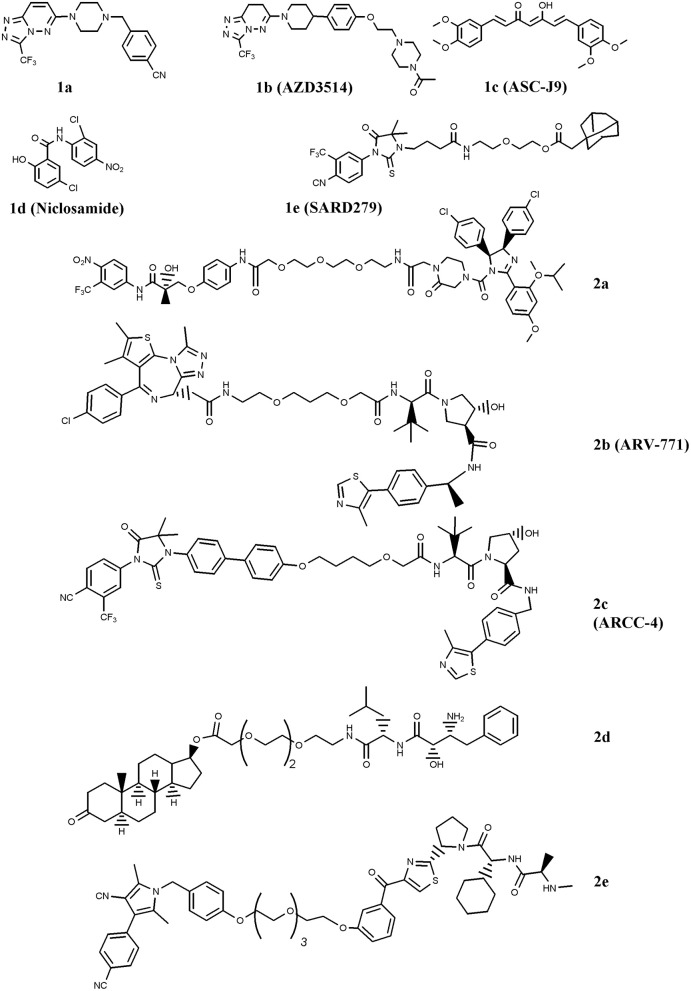
Chemical structures of AR-ligand based conjugates that reduce AR expression. The chemical structures of Selective AR degraders (SARD, **1a–e**) and PROteolysis TArgeting Chimeras (PROTAC, **2a–e**) are reported.

The first SARD reported is the benzyl-piperazine derivative **1a**, Figure 1 (Bradbury et al., 2011). The compound contains a cyanobenzyl moiety that favors the interaction with AR, and resulted from a two-dimensional pharmacophore modeling which correlates the binding of diidrotestosterone (DHT) and bicalutamide to AR compared with a series of benzyl-piperazine derivatives. The molecule caused moderate receptor down-regulation in LNCaP cells, and was supposed to produce adverse cardiovascular effects due to a predicted potentiation of the hERG ion channel. This drawback has been tackled by using a comprehensive medicinal chemistry optimization programme which led to compound AZD3514 (**1b**), Figure 1 (Bradbury et al., 2013). The dihydrotriazolopyridazine derivative is devoid of hERG effect, impairs ligand-driven nuclear translocation of the receptor and down-regulates AR *in vitro* and *in vivo* (Bradbury et al., 2013; Loddick et al., 2014). Two phase I clinical studies (NCT01351688 and NCT01162395; www.clinicaltrials.gov) have been completed in metastatic CRPC patients. In spite of the important side effects observed (nausea and vomiting), patients treated with AZD3514 showed significant Prostate-Specific-Antigen (PSA) reduction and disease stabilization (Cummings et al., 2014; Omlin et al., 2015).

Another SARD is ASC-J9 (**1c**), Figure 1 (Guo et al., 2017). This molecule showed interesting capability to degrade both wild-type (wt) AR and Androgen-Receptor-splice-Variant 7 (AR-V7), a receptor variant found in ADT-treated patients that lacks ligand-binding domain (Yamashita et al., 2012; Wang et al., 2017). Furthermore, Wang et al. (2016a) reported the interesting activity of ASC-J9 on AR-F876L mutant, a condition found in enzalutamide-resistant CRPC patients. More recently, in drug combination studies, the compound was reported to counteract AR enhancement observed in CRPC cells exposed to docetaxel, and consequently increase docetaxel sensitivity. This finding suggests a possible drug combination strategy for docetaxel-resistant PC patients (Luo et al., 2018). Additionally, ASC-J9 was found to negatively impact on PC proliferation and invasion by perturbing, via AR reduction, AR-STAT3-CCL2 /CCL3 /CCL4, AR/FANS as well as AR-p62 axis (Jiang et al., 2012; Wang et al., 2012; Fang et al., 2013; Lin et al., 2013, 2018; Wen et al., 2016). Based on these results, ASC-J9 has been selected for clinical investigations and recently Food-and-Drug-Administration (FDA) approved different solvents for compound formulation (Cheng et al., 2018)

A significant reduction of AR-V7 level is observed in PC cells treated with niclosamide (**1d**) (Figure 1), a drug approved by FDA for anti-helminthic therapy (Elshan et al., 2018; Sobhani et al., 2018). By reducing AR-V7, niclosamide inhibits receptor transcriptional activity and blocks its recruitment on the PSA promoter region (Liu et al., 2014). The significant tumor growth reduction observed in enzalutamide- or abiraterone acetate-insensitive xenograft bearing mice treated with the drug combinations niclosamide/enzalutamide or niclosamide/abiraterone acetate suggested possible drug combination interventions for enzalutamde- or abiraterone acetate-resistant CRPC patients (Liu et al., 2014, 2016, 2017). Two phase I trials (NCT03123978 and NCT02532114) including niclosamide and enzalutamide, and one phase II clinical study (NCT02807805) combining niclosamide with abiraterone acetate have been activated (www.clinicaltrials.gov). Results of the phase I study recently concluded indicate that, due to the toxicity observed, niclosamide is not a suitable drug for repurposing as a CRPC treatment (Schweizer et al., 2018).

SARD also includes a class of compounds containing a hydrophobic residue (e.g., chemical degrons) coupled with an AR ligand. The hydrophobic moiety mimics a partially denatured protein state (hydrophobic tagging) that recruits chaperons and in turn induces proteasome-mediated degradation of the receptor (Lai and Crews, 2017). Among the chemical degrons considered, adamantane group was found very interesting, and the conjugation of an adamantane moiety with the AR agonist RU59063 resulted in SARD279 (**1e**) (Figure 1) (Toure and Crews, 2016). In spite of the 37-fold reduction in AR binding affinity observed for the conjugate compared with parent RU59063, SARD279 reduced receptor levels (including AR-F876L variant) and in turn the expression of AR-related genes. This finding is corroborated by the capability of the compound to overcome enzalutamide resistance in CRPC cells (Gustafson et al., 2015).

### PROteolysis Targeting Chimeras (PROTAC)

Very interesting for PC therapy is PROTAC, a class of compounds containing two recruiting ligands: a E3-ubiquitin ligase binding moiety and a AR binder. By interacting with AR, these chimeras direct the ligase activity on the exposed lysines of the receptor and favor ubiquitination/polyubiquitination and in turn its proteasome-mediated degradation (Churcher, 2018). The first PROTAC engineered for fighting PC merges the E3-ubiquitin ligase MDM2 ligand nutilin with a non-steroidal AR binder via a Polyethylene-Glycol (PEG) linker (**2a**) (Figure 1) (Schneekloth et al., 2008). The proof of principle that the compound activates the protesome-mediated degradation of AR is the observation that no down-regulation of the receptor, and consequently no compound activity, was observed in cells pretreated with a proteasome inhibitor (Toure and Crews, 2016).

Another family of proteins considered for PROTAC strategy is the bromodomain and extraterminal (BET) proteins, e.g., BRD 2, 3, and 4. These proteins are AR co-activators that activate receptor-related functions. Compounds that disrupt AR-BRD interaction by inducing BRD degradation impair PC growth (Raina and Crews, 2017). A molecule endowed with this function is ARV-771 (**2b**) (Figure 1) (Raina et al., 2016). The compound contains a von Hippel-Landau E3-ligase moiety coupled with a selective BRD binder, namely JQ1. ARV-771 attenuates AR signaling of both wt and AR-V7 receptor variant, reduces the levels of BRD proteins, and activates apoptosis via Poly-ADP-Ribose-Polymerase (PARP) cleavage and caspase 3/7 activation. Moreover, ARV-771 reduces CRPC cells proliferation *in vitro* and induces antitumor activity in CW22Rv1 and VCaP tumor xenograft models. In this context, Salami et al. reported the chimera ARCC-4 (**2c**), a compound containing the von Hippel-Landau E3-ligase moiety coupled with enzalutamide for AR targeting (Salami et al., 2018). Compared with enzalutamide, ARCC-4 better down-regulates AR, including mutant forms (F876L, T877A, L702H, H874Y, M896V), and is more active in reducing cell proliferation and in inducing caspase 3/7-mediated apoptosis in CRPC cell lines.

Specific and Non-genetic Inhibitor-of-Apoptosis proteins (IAPs)-dependent Protein Eraser (SNIPER) is a peculiar type of PROTAC which contains a IAP ligand functioning as an E3-ubiquitin ligase binder (Lai and Crews, 2017). The SNIPER by Itoh et al. (2011) coupled bestatin (IAP ligand) with a steroidal AR binder (**2d**, Figure 1). The compound significantly reduced AR levels in AR-expressing breast cancer MCF7 cells. Very recently, another SNIPER based on a different IAP ligand (LCL-161) conjugated with a non-steroidal AR antagonist has been reported by Shibata et al. (2018), **2e**. The compound, ineffective on AR-independent PC3 cells, markedly reduced the cell growth of AR-dependent (VCaP and LNCaP) cell lines. Effective and specific proteasome-mediated knockdown of AR and apoptotic cell death (PARP and caspase 3 cleavages) have been reported in VCaP cells treated with **2e**. These findings paralleled with the reduced expression of receptor-associated genes (PSA, TMPRSS2, KLK2, and NKX3.1), and this behavior reverted when cells were pretreated with the proteasome inhibitor MG132.

## AR-Ligand Based Conjugates for Targeting Small-Molecules

It is well-known that conventional antitumor drugs fail to select tumor tissues and this feature causes important side effects often requiring treatment suspension. Aimed at targeting small-molecules, including conventional chemotherapeutics, toward AR-expressing PC cells, several compounds have been conjugated with AR binder.

### Conjugates for Targeting Conventional Chemotherapeutics

**Figure 2:**
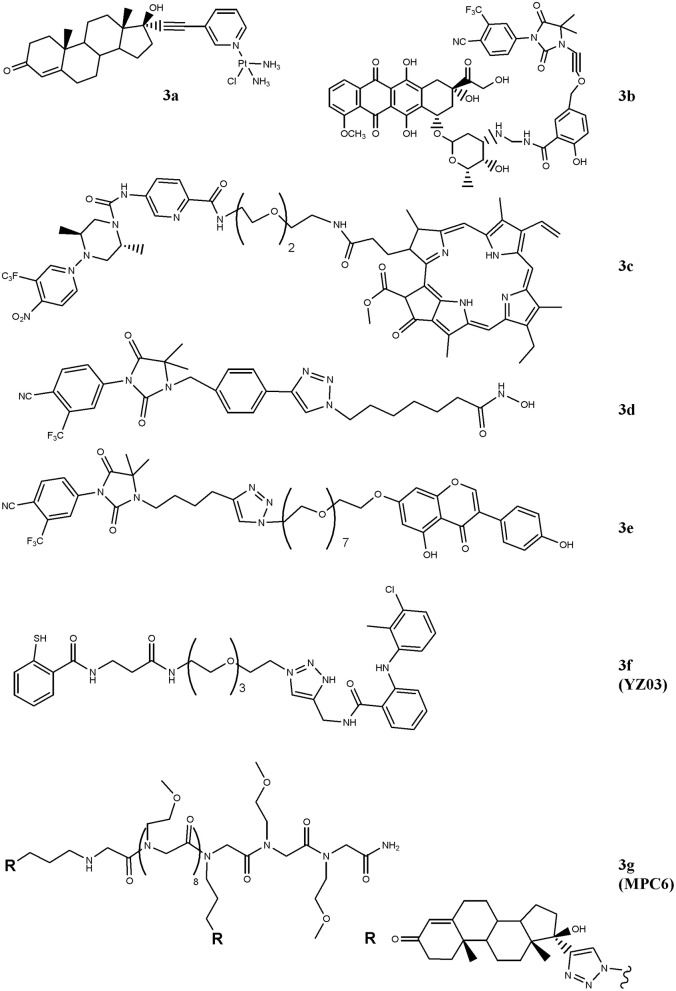
Chemical structures of AR-ligand based conjugates for targeting small-molecules. The hybrid compounds containing conventional antitumor drugs (cisplatin, **3a**; doxorubicin, **3b**; and pheophorbide α, **3c**) are reported. The figure also shows the chemical structures of molecules that alter chromatin status (**3d** and **3e**) and that of compounds that interfere with AR/co-activators interactions (**3f** and **3g**).

In order to drive cisplatin (e.g., formation of platinum-DNA adducts) against PC cells, the drug has been conjugated with the testosterone-homolog ethisterone, **3a** (Figure 2) (Huxley et al., 2010; Sanchez-Cano et al., 2010). By using a modified Sonogashira cross-coupling reaction, the ethisterone has been coupled with different bromo substituted nitrogen-based heterocycles, including pyridine, quinolone and isoquinoline, carrying a platinum unit. Among the compounds designed, only those containing the pyridine linker were obtained in good amount and resulted cytotoxic for PC cells. The best derivative of the series (**3a**) preferentially accumulated in AR-positive cell lines and this finding paralleled with the improved cytotoxicity observed in these cells in comparison with AR-negative cells (Huxley et al., 2010).

Similarly, doxorubicin, a well-studied and potent topoisoemarse II poison, has been considered (Cogan and Koch, 2004). The conjugate generated by Cogan and Koch contains doxaliform, a doxorubicin-derivative obtained condensing doxorubicin with salicylamide (2-hydroxybenzamide) and formaldehyde, which is more cytotoxic than doxorubicin on doxorubicin-sensitive and -resistant tumor cells. To target doxaliform on AR-expressing cells, an alkyl-cyano nilutamide moiety has been attached to doxaliform via a linear alkyne cleavable tether (**3b**) (Figure 2). The conjugate binds AR and, after receptor-mediated nuclear translocation, releases doxaliform in cytotoxic amount. This behavior was observed in AR-transfected but not in untransfected PC3 cells.

A very intriguing way to pursue for anticancer therapy is the development of compounds that, after light activation, selectively release nitric oxide (NO) into cancer cells (Rapozzi et al., 2015, 2017) paved this way by designing a bi-functional compound (**3c**, Figure 2) containing a dimethyl-piperazine moiety substituted with a trifluoromehylnitrobenzene ring (NO_2_-modified AR binder) coupled with a pheophorbide α moiety, which allows the production of reactive oxygen species, after light exposure. The compound is totally safe in the dark and following exposure to white light both pheophorbide α and NO donor moieties are activated. The molecule showed nuclear localization in CRPC and, differently to pheophorbide α alone, it by-passes the ABCG2-mediated efflux, thus making this compound interesting for patients resistant to photodynamic therapy. Gaining further insight into compound's action mechanism, the same research group identified the doubtful role played by the NF-kB/YY1/RKIP pathway in mediating NO activity of the conjugate. Specifically, NO mediates pro- or anti-survival activity depending on concentration levels achieved following light activation. Low NO levels activate a pro-survival/anti-apoptotic NF-kB/YY1 pathway; conversely, high NO release inhibits NF-kB/YY1 and, via Snail, activates the anti-survival/pro-apoptotic RKIP leading to antitumor activity. Thus, the two photosensitive moieties synergize and induce cell death following conditions that produce high NO levels.

### Conjugates for Chromatin Remodeling

HDAC-containing therapy is among medical interventions proposed for the treatment of patients suffering from cancer, including PC (Sonnenburg and Morgans, 2018). These drugs inhibit histone deacetylases, a class of enzymes that removes acetyl groups from ε-N-acetyl lysines on histone proteins allowing chromatin decondensation. This activity impacts on gene expression, tumor growth and drug resistance (Graça et al., 2016). Although very effective in preclinical models, these drugs did not improve survival of patients affected by solid tumors, likely dependent, at least in part, on their reduced cellular accumulation (Gryder et al., 2013). Aimed at implementing drug uptake in PC cells, Gryder and co-workers designed a hybrid HDAC inhibitors equipped with a non-steroidal (cyano-nilutamide) AR binder. Specifically, an aryl-alkyne or alkyl-alkyne cyano-nilutamide moiety has been conjugated, via a triazole alkyl linker, with the zinc chelating hydroxamate residue. Compared with alkyl-alkyne derivatives, compounds belonging to the aryl-alkyne series resulted more active. Derivative **3d** (Figure 2) showed the best interaction with AR and maintained HDAC activity. Cyano-nilutamide moiety increased nuclear localization of HDAC inhibitory activity in androgen-dependent LNCaP cells and in less extent in castration-resistant DU145 cells.

The status of the chromatin is also controlled by histone acetyltransferase (HAT) enzymes. These enzymes catalyze acetylation of histones and produce chromatin decondensation allowing changes in gene expression and protein levels that in turn impact on cellular response to antitumor drugs. By increasing HAT levels, genistein increases p21 and p16 expression and favors cell-cycle arrest and apoptosis. Aimed at directing genistein action against AR-expressing PC cells, bi-functional agent containing a non-steroidal moiety linked to genistein (hydantoin-derived antiandrogen-genistein) has been recently designed (**3e**), Figure 2 (George et al., 2018). The compound is more active than genistein and enzalutamide administered alone in inducing S-phase cell-cycle arrest and in reducing cell proliferation of LNCaP, DU145, and CW22Rv1 cells. The hybrid molecule also down-regulates the expression of AR through the inhibition of HDAC6-Hsp90 co-chaperone function.

### Conjugates That Impair AR/Co-activators Interaction

The interaction of AR with co-activators critically regulates receptor functions and compounds that interfere with this binding impact on tumor growth. In this regard, two hybrid derivatives containing a AR binder and endowed with AR/co-activator interfering action (YZ03, **3f**, and MPC6, **3g**) have been reported (Wang et al., 2016b; Zhang et al., 2016). The acetyl-transfer activity of thiosalycilamides has been directed toward AR by conjugating tolfenamic acid (AR binder) with the thiosalycilamide (Figure 2) (Zhang et al., 2016). YZ03 increases the acetylation of Lys720 of AR in CW22Rv1 cells. This amino acid is critical for the binding of AR to co-activators and its acetylation produces a steric hindrance that negatively impacts on AR/co-activators binding. No data are available about the cytotoxic activity of YZ03. Another molecule that interferes with AR/co-activator interaction is the multivalent peptoid conjugate MPC6 (**3g**). This compound consists of two ethisterone moieties (AR binder) linked each other via a peptoid oligomer (Figure 2) (Wang et al., 2016b). The interaction of MPC6 with AR blocks, by steric clash, the binding of the receptor with co-activators and reduces the expression of both wt and AR-V7 forms of AR. The compound reduces the proliferation *in vitro* of AR-expressing PC cells (LNCaP), including those resistant to bicalutamide (LNCaP-C4-2) and enzalutamide (LNCaP-abl; LNCaP-95). Noteworthy, MPC6 showed favorable pharmacologic profile and antitumor potency *in vivo* against enzaltamide-resistant LNCaP-abl tumor xenografts.

## Conclusions

AR is an interesting player to engineer receptor-directed conjugates for targeting PC. Directed by AR-ligand, these compounds produce selective AR down-regulation and/or favor nuclear accumulation of chemotherapeutics. These conjugates are difficult to design since they have to challenge several issues to maintain the “druggability,” including (i) the proneness of the two pharmacophores to tolerate chemical modifications without affecting the interaction with the targets; (ii) the physical-chemical properties (size and solubility) of the two moieties, which are fundamental for compound formulation; and (iii) linker length and flexibility, which provide optimal distance between the two pharmacophores and allow them to adapt on the targets. In this regard, ligand-based and/or structure-based computational approaches are very useful for the design and optimization of hybrid molecules.

Some of the conjugates reported (e.g., **1b**, **1c**, **1d**, **1e**, **2b**, **2c**, **3e**, and **3g**) have been tested on CRPC and proved efficacy in overcoming drug resistance developed against AR-targeted drugs. Compounds that function by reducing the levels of the receptor are in principle very interesting for patients resistant to ADT due to AR mutations. Indeed, these molecules activate proteasome-mediated degradation of both wt and AR mutant variants. In this context, SARD (**1b**, **1c**, **1d**, and **1e**) and PROTAC (**2b** and **2c**) proved efficacy on CRPC.

It is important to underline that the synthesis of hybrid molecules may result in high molecular weight conjugates (>1,000 Da). According to Lipinski's rule of five (Lipinski et al., 2001), this feature negatively impacts on drug-like property of molecules. In this regard, it is noteworthy that among the compounds described, only **1b** and **1d** (e.g., low molecular weight compounds) progressed beyond the biochemical/cellular characterization and the clinical setting. Unfortunately, the results of clinical trials conducted so far are not encouraging because of important side effects experienced by patients.

In conclusion, although the compounds here reported may be considered a sort of next-generation AR-targeted drugs, most of them represent proof of concept supporting the feasibility of a pharmacological strategy rapidly evolving and arousing great interest. The benefits for patients suffering from PC have yet to be proven.

## Author Contributions

All authors listed have made a substantial, direct and intellectual contribution to the work, and approved it for publication.

### Conflict of Interest Statement

The authors declare that the research was conducted in the absence of any commercial or financial relationships that could be construed as a potential conflict of interest.
